# Characterization of the Immune Response of MERS-CoV Vaccine Candidates Derived from Two Different Vectors in Mice

**DOI:** 10.3390/v12010125

**Published:** 2020-01-20

**Authors:** Entao Li, Feihu Yan, Pei Huang, Hang Chi, Shengnan Xu, Guohua Li, Chuanyu Liu, Na Feng, Hualei Wang, Yongkun Zhao, Songtao Yang, Xianzhu Xia

**Affiliations:** 1 College of Veterinary Medicine, South China Agricultural University, Guangzhou 510642, China; liet0706@163.com; 2 Key Laboratory of Jilin Province for Zoonosis Prevention and Control, Changchun Veterinary Research Institute, Chinese Academy of Agricultural Sciences, Changchun 130122, China; yanfh1990@gmail.com (F.Y.); zhaoyongkun1976@126.com (Y.Z.); 3 Animal Science and Technology College, Jilin Agricultural University, Changchun 130118, China; 4 College of Animal Science and Technology, Shihezi University, Shihezi 832003, China; 5 College of Veterinary Medicine, Jilin University, Changchun 130062, China; 6 Jiangsu Co-Innovation Center for the Prevention and Control of Important Animal Infectious Disease and Zoonosis, Yangzhou University, Yangzhou 225009, China

**Keywords:** MERS-CoV, recombinant rabies virus, bacterium-like particle, immune response

## Abstract

Middle East respiratory syndrome (MERS) is an acute, high-mortality-rate, severe infectious disease caused by an emerging MERS coronavirus (MERS-CoV) that causes severe respiratory diseases. The continuous spread and great pandemic potential of MERS-CoV make it necessarily important to develop effective vaccines. We previously demonstrated that the application of Gram-positive enhancer matrix (GEM) particles as a bacterial vector displaying the MERS-CoV receptor-binding domain (RBD) is a very promising MERS vaccine candidate that is capable of producing potential neutralization antibodies. We have also used the rabies virus (RV) as a viral vector to design a recombinant vaccine by expressing the MERS-CoV S1 (spike) protein on the surface of the RV. In this study, we compared the immunological efficacy of the vaccine candidates in BALB/c mice in terms of the levels of humoral and cellular immune responses. The results show that the rabies virus vector-based vaccine can induce remarkably earlier antibody response and higher levels of cellular immunity than the GEM particles vector. However, the GEM particles vector-based vaccine candidate can induce remarkably higher antibody response, even at a very low dose of 1 µg. These results indicate that vaccines constructed using different vaccine vector platforms for the same pathogen have different rates and trends in humoral and cellular immune responses in the same animal model. This discovery not only provides more alternative vaccine development platforms for MERS-CoV vaccine development, but also provides a theoretical basis for our future selection of vaccine vector platforms for other specific pathogens.

## 1. Introduction

Middle East respiratory syndrome coronavirus (MERS-CoV) was first identified in Saudi Arabia in 2012 [1]. Soon after its first report, some imported cases occurred in many other countries outside the Arabian Peninsula through infected travelers, most notably the outbreak in South Korea in 2015 [2]. The clinical signs of MERS-CoV-infected patients are similar to Severe Acute Respiratory Syndrome coronavirus (SARS-CoV), the outbreaks of which in China in 2003 caused acute respiratory distress syndrome in humans [3]. Bats and dromedaries are considered as the natural reservoirs of MERS-CoV [3]. Up to now, a continuous increase of human cases in the endemic areas has been reported [4], highlighting that it is urgent to develop and make available effective therapeutic drugs and prophylactic vaccines to protect against MERS-CoV.

To date, many promising approaches toward MERS-CoV vaccine candidates have been developed, such as recombinant viral vectors [4,5,6,7,8,9,10], protein-based platforms [11,12], bacterial-based platform [13], and DNA vaccines [14,15,16]. Most of MERS-CoV vaccine candidates have focused on the major immune-dominant antigen, the S protein of the virion, which plays a significant role in mediating viral entry into target cells and in inducing neutralizing antibodies in experimental animals and infected individuals [17]. The receptor-binding domain (RBD) within the S1 domain of the S protein of the MERS-CoV virus decides the host range and cellular tropism [18,19,20]. As such, we developed two promising MERS-CoV vaccine candidates using two different platforms. One uses the rabies virus (RV) as a vector to express S1 domains of the MERS-CoV S gene to develop a recombinant MERS-CoV vaccine, and the other applies gram-positive enhancer matrix (GEM) particles as a vector to construct a MERS bacterium (*Lactococcus lactis*)-like particle (BLP) vaccine by displaying the RBD [13]. A previous study has utilized the RV (BNSP333) vector modified from the RV vaccine vector SPBN to successfully design a vaccine against MERS-CoV infection [10,21], whereas the RV vector (SRV9 strain) used in our study is screened from the Street Alabama Dufferin (SAD) strain by plaque purification on baby Syrian hamster kidney cells (BHK-21) cells, which have been proven to be safe and have good immunogenicity [22]. The GEM-PA system is a novel surface display system consisting of nonliving and genetically unmodified GEM particles and a protein anchor (PA) from the *Lactococcus lactis* peptidoglycan hydrolase AcmA. We utilized the system to successfully display the RBD protein fragment of MERS-CoV, inducing high neutralizing antibodies with intramuscular vaccination and robust antigen-specific local and systemic immune responses by intranasal vaccination [13]. The head-to-head comparison of available vaccine vectors is necessary in vaccine design [23]. The difference in immune response induced by two different MERS-CoV vaccine candidates and the effect of antigen displayed in viral and bacterial vectors on the immune response are still questionable.

In this study, we evaluated the immunological efficacy of MERS BLP at different doses and the inactivated recombinant MERS antigen in BABL/c mice. Specifically, we compared the ability of these two vaccines to induce MERS-CoV-specific humoral immune response and T-cell-mediated immune response, as well as neutralizing antibodies against MERS-CoV.

## 2. Materials and Methods

### 2.1. Ethics Statement

All animal works were strictly in accordance with the welfare and ethical guidance on Chinese laboratory animals (GB 14925-2001). The agreement was approved by the Animal Welfare and Ethics Committee of the Institute of Veterinary Medicine of the Changchun Veterinary Research Institute (Laboratory Animal Care and Use Committee Authorization, permit number JSY-DW-2019-05).

### 2.2. Cells and Virus

Baby Syrian hamster kidney (BHK-21) cells were cultured in serum-free medium (VirusPro^®^, Shanghai, China) at 37 °C with 5% CO_2_ on an orbital shaker at 130 rpm in suspension culture for virus infection. Mouse neuroblastoma (NA) cells were cultured with Dulbecco’s modified eagle medium (DMEM, Thermo Fisher Scientific, Waltham, MA, USA) plus 10% fetal bovine serum (FBS, Biological Industries, Kibbutz Beit Haemek, Israel) for determination of viral titer and verification of virus inactivation. *Spodoptera frugiperda* 9 (Sf9, Gibco, Grand Island, NY, USA) insect cells were grown in Sf-900TM II medium (Life Technologies, San Diego, CA, USA) for protein expression. The recombinant RV expressing MERS-CoV S1 protein (RV/MERS) was constructed and stored in our laboratory.

### 2.3. Production of the RV/MERS in BHK-21 Cells

The RV/MERS virus was cultivated in BHK-21 cells. Briefly, BHK-21 cells (2 × 10^6^ cells per mL) grown in shake flask cultures were infected with RV/MERS virus at a multiplicity of infection (MOI) of 0.05. RV/MERS was harvested from the culture supernatant of cell culture on two days post-infection (DPI). The titers of the recombinant virus were determined by the median endpoint of the 50% tissue culture infectious dose units (TCID_50_) in NA cells as described previously [24]. Recombinant virus was inactivated with 0.025% β-propiolactone (*v*/*v*); the mixtures were incubated at 4 °C overnight and then treated at 37 °C for 2 h. The verification of the virus’ complete inactivation was detected in NA cells. The completely inactivated virus was purified with 500KD Hollow Fiber Ultrafiltration Column and Sepharose Fast Flow 4 Gel. Fractions were harvested and analyzed by immunoblotting. The total protein concentration of the purified product was determined by the bicinchoninic acid (BCA) protein assay.

### 2.4. Production of the MERS Bacterium-Like Particles

The MERS BLP was produced as previously described [13]. Briefly, the fusion protein of RBD-linker-PA3 was expressed in a baculovirus expression system. The GEM particles were used as vectors to construct MERS BLP by externally displaying the RBD through the PA3. The fusion protein concentration of the MERS BLP was detected densitometrically using Quantity One image analysis software, version 4.6.7.

### 2.5. Size of the Particles

The size (Hydrodynamic diameter, in nm) and size distribution (polydispersity index, PDI) of the RV/MERS and MERS BLP were determined using a laser particle size analyzer (NANO ZS90, Malvern Instruments, Worcestershire, UK), which the fresh prepared RV/MERS and MERS BLP suspension were determined under almost the same humidity and temperature at 25 °C, and each sample measurement was performed in triplicate simultaneously.

### 2.6. Animal Immunization and Sera Collection

Four- to six-week-old female BALB/c mice were purchased from Changchun Yisi Laboratory Animal Technology Co., Ltd. (Changchun, China). Female BALB/c mice were randomized into five groups of 10. Mice were intramuscularly prime-immunized with 8 µg purified RV/MERS virus; 1, 5, and 20 µg MERS BLP; mixed with the compound adjuvants consisting of ISA201VG (Seppic, Paris, France) and Poly I: C (Sigma, St. Louis, MO, USA); and boosted twice with an equal volume antigen as the primary vaccination at 3-week intervals. Compound adjuvants only were immunized as negative control. Blood samples were collected before immunization and two weeks after each vaccination. Serum samples were inactivated at 56 °C for 30 min before determining MERS-CoV RBD-specific antibodies and neutralizing antibodies. Five mice selected randomly from each group were euthanized on day 7 after the third immunization, and their splenocytes were collected for cytokines analysis.

### 2.7. ELISA for MERS-CoV Specific Antibody Test

Enzyme-linked immunosorbent assay (ELISA) was applied to analyze MERS-CoV RBD-specific IgG, IgG1, IgG2a, IgG2b, IgG2c, and IgG3 antibodies. Serially diluted sera were added to 96-well microtiter plates (Corning-Costar, Corning, NY, USA) that were pre-coated overnight with MERS-RBD protein (1 μg/mL) produced in *Escherichia coli*. The plates were incubated at 37 °C for 1 h and washed three times with PBST before being incubated with the following horseradish peroxidase (HRP) labeled anti-mouse IgG (1:5000, BioWorld, St. Louis, MN, USA), IgG1, IgG2a, IgG2b, IgG2c, and IgG3 (1:2000, Southern Biotech, Birmingham, AL, USA) at 37 °C for 1 h. Plates were then washed three times, and 100 µL TMB (3′,3′,5′,5′-tetramethylbenzidine) per well was added. Then, the color development was stopped by adding H_2_SO_4_. Optical density values were read at 450 nm using an ELISA plate reader (Bio-Rad, Hercules, CA, USA).

### 2.8. Virus Neutralizing Assay

Neutralizing titers of mice sera were detected by using a MERS-CoV pseudovirus reported in our previous study [11]. Briefly, serially diluted mouse sera mixed with an equal volume of 100× TCID_50_ of MERS-pseudotyped viruses were incubated at 37 °C for 30 min, and then, the mixture was incubated with Huh 7 cells at 37 °C for 4 h. After the incubation, the medium was replaced with complete DMEM containing 10% fetal bovine serum and 1% penicillin–streptomycin, and then, the samples were incubated at 37 °C for 48 h. The luciferase activity was detected by using an Infinite M200 Microplate Spectrophotometer (Tecan, Männedorf, Switzerland).

### 2.9. Cytokines Analysis in Splenocytes Culture Supernatants

To evaluate antigen-specific T-cell responses, splenocytes were harvested on day 7 after the third immunization and stimulated with 1 μM MERS-CoV peptide S291 for 24 h at 37 °C [25]. Secreted tumor necrosis factor-alpha (TNF-α), interferon-gamma (IFN-γ), and interleukin 2 (IL-2) in the supernatants were detected with the mouse ELISA cytokine kits (MABTECH, Nacka, Sweden).

### 2.10. Statistics

All statistical analysis was performed by using the Graphpad 8.0.2 software. The results are expressed as the mean ± SD, and significance in their differences between groups were analyzed using a Student’s *t*-test.

## 3. Results

### 3.1. The Particle Size of RV/MERS and MERS BLP

**Figure 1:**
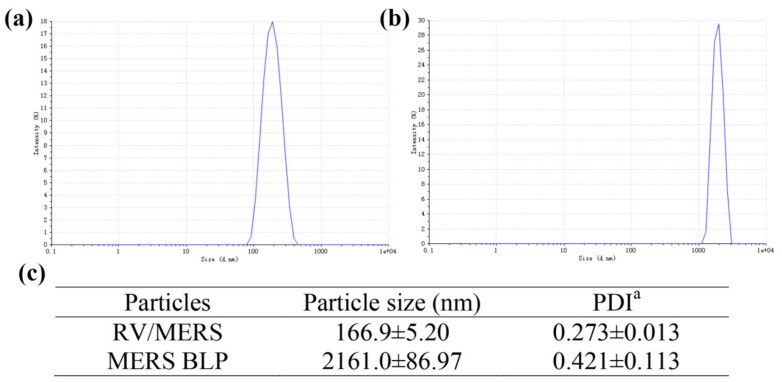
The particle size of recombinant RV expressing MERS-CoV S1 protein (RV/MERS) and MERS bacterium-like particle (MERS BLP). The particle size distribution of RV/MERS (**a**) and MERS BLP (**b**). The particle size and polydispersity index (PDI) of RV/MERS and MERS BLP (**c**). Data was shown as mean ± SD (*n* = 3). ^a^ PDI, polydispersity index from dynamic light scattering (DLS).

The particle size and polydispersity index of RV/MERS and MERS BLP were measured by a dynamic light scattering (DLS) device before immunization of mice. As shown in Figure 1, the size of RV/MERS is about 167 nm (Figure 1a); the size of MERS BLP is about 2161 nm (Figure 1b).

### 3.2. Neutralizing Antibody Response by Pseudovirions Neutralization Assay

**Figure 2:**
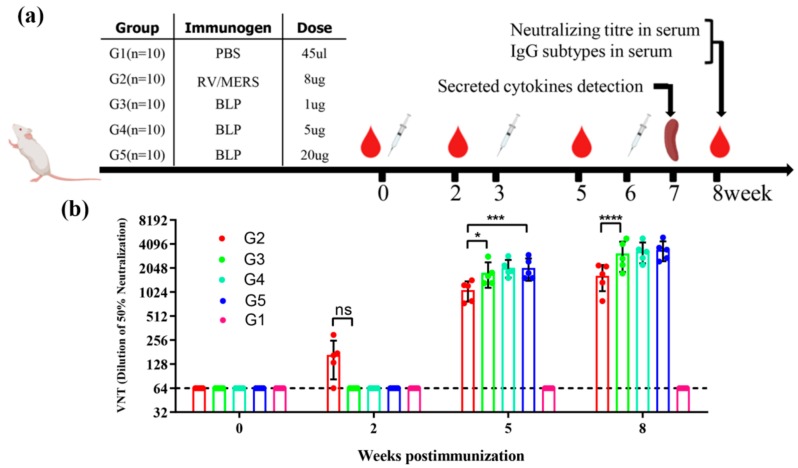
Schematic diagram of immunization and neutralizing antibody responses. (**a**) The vaccination schedule and characterization of immunologic responses in BALB/c mice. A total of five groups of mice were immunized and detected for virus-neutralizing antibody, IgG subtypes, and cytokines release. Group 1 (G1) was negative controls; group 2 (G2) was vaccinated with inactivated and purified rabies virus (RV)/Middle East respiratory syndrome (MERS); group 3, group 4, and group 5 (G3, G4, G5) were vaccinated with varying doses of the MERS bacterium (*Lactococcus lactis*)-like particle (BLP). All groups received a second and third identical vaccination boost with a combined adjuvant at 3-week intervals after the primary immunization. (**b**) Neutralizing activity was detected by using MERS coronavirus (MERS-CoV) pseudovirus, and the data are shown as the mean ± SD from five mice in each group and were analyzed by Student’s *t*-test (* *p* < 0.05, *** *p* < 0.001, **** *p* < 0.0001, *n* = 5).

To compare the immunological efficacy of two MERS-CoV vaccines derived from two different vectors, we studied the impact on the immunogenicity of MERS-CoV BLP with different doses and the immunogenicity of RV/MERS with only one certain dose in mice. The schematic diagram for group design, immunizations, and immunological characterization is shown in Figure 2a. A total of five groups of mice (G1–G5) were administered three immunizations with a combined adjuvant through an intramuscular (i.m.) route; G1 was immunized with PBS as negative control; G2 was immunized with 8 µg inactivated and purified RV/MERS; G3–G5 were immunized with 1 µg (low dose), 5 µg (medium dose), and 20 µg (high dose) MERS-CoV BLP, respectively. Sequential sera samples were collected at weeks 0, 2, 5, and 8 and detected for neutralizing activity against pseudotyped MERS-CoV and the binding activity. Splenocytes were harvested at 7 weeks after the last immunization for cytokines analysis. As shown in Figure 2b, G2 developed neutralizing antibody response at 2 weeks after the first immunization, and the neutralizing antibodies of G3, G4, and G5 were not detected at this point. However, the neutralizing antibody of G3, G4, and G5 was significantly higher than G2 at 5 and 8 weeks after immunization. There were also no significant differences in the neutralizing activity among G3, G4, and G5, indicating that 1 µg MERS-CoV BLP can efficiently induce MERS-CoV neutralizing antibodies in mice. Overall, these results show that the RV/MERS can rapidly induce neutralizing antibody response, and the MERS BLP can induce higher antibody response in mice.

### 3.3. MERS-CoV RBD-Specific IgG Subtypes Responses by Indirect ELISA

**Figure 3:**
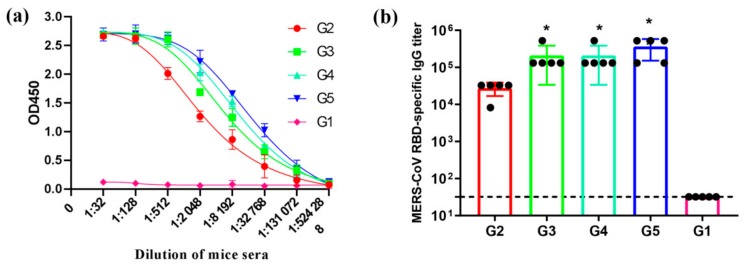
MERS-CoV receptor-binding domain (RBD)-specific IgG antibodies. Sera from 2 weeks after the last immunization were tested for MERS-CoV RBD-specific IgG antibodies by enzyme-linked immunosorbent assay (ELISA). The data are shown as the mean ± SD from five mice in each group and were analyzed by Student’s *t*-test (* *p* < 0.05, n = 5). (**a**) The total IgG antibodies specific to MERS-CoV RBD protein were assessed by ELISA. The level of the MERS-CoV RBD-specific IgG (**b**) antibodies was determined by ELISA and is shown as end-point dilution titers.

We next detected the MERS-CoV RBD-specific IgG and IgG subtypes’ antibodies level in the serum at 8 weeks post-immunization. ELISA results indicate that both MERS antigen-induced IgG antibodies can specifically bind to MERS-CoV RBD protein. Particularly, G3–G5 acquired a stronger binding to MERS-CoV RBD protein than G2, whereas G1, the control samples, were detected to have negligible binding activity (Figure 3a), indicating the specificity of the IgG antibody response induced by this RBD protein. Additionally, the endpoint titers of the IgG antibody level showed that the immunogenicity of G3–G5 was much stronger than G2 in inducing MERS-CoV RBD-specific IgG antibody response in mice after three vaccinations (Figure 3b). Furthermore, no significant difference was shown for IgG titers among groups G3, G4, and G5, suggesting that 1 µg antigen protein on the bacterial vector is sufficient to induce high RBD-specific antibody level in mice. Thus, the MERS-CoV antigen protein displayed on the surface of the bacterial vector can significantly enhance the immunogenicity of the RBD fragments.

**Figure 4:**
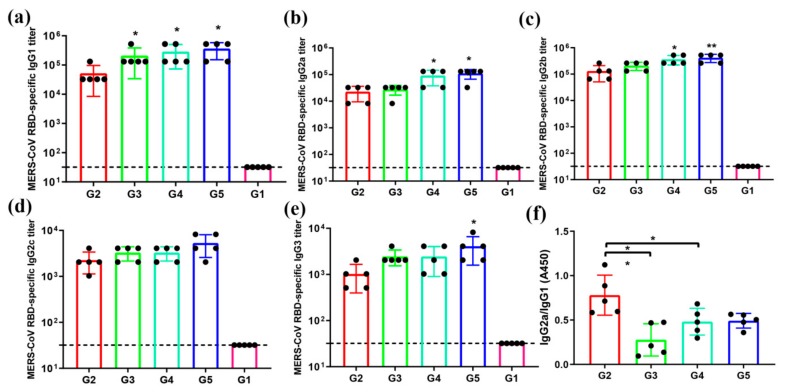
MERS-CoV RBD-specific antibody subtypes response. Sera from 2 weeks after the last immunization were tested for MERS-CoV RBD-specific IgG, IgG1, IgG2a, IgG2b, IgG2c, and IgG3 antibodies by ELISA. The data are shown as the mean ± SD from five mice in each group and were analyzed by Student’s *t*-test (* *p* < 0.05, ** *p* < 0.01, *n* = 5). The level of the MERS-CoV RBD-specific IgG1 (**a**), IgG2a (**b**), IgG2b (**c**), IgG2c (**d**), and IgG3 (**e**) antibodies were determined by ELISA and are shown as end-point dilution titers. The ratios between IgG1 and IgG2a antibody responses were also calculated (**f**).

T helper 2 (Th2) response is associated with IgG1, and T helper 1 (Th1) response correlates with IgG2a, IgG2b, IgG2c, and IgG3 in mice [26]. In order to evaluate the IgG subtypes’ levels in serum from immunized mice, the production of IgG1, IgG2a, IgG2b, IgG2c, and IgG3 in sera were detected. Observation of sera IgG subtypes showed that the sera in G3–G5 induced significantly higher levels of IgG1 antibody than those of G2 (Figure 4a); the sera in G4–G5 induced significantly higher levels of IgG2a and IgG2b antibodies than those of G2 (Figure 4b,c); there was no significant difference in IgG2c antibody level between G2–G5 (Figure 4d); the sera in G5 induced significantly higher levels of IgG3 antibody than those of G2 (Figure 4e). However, further analysis of the ratios of IgG2a/IgG1 revealed that the sera in G2 were remarkably higher than those in G3–G5 (Figure 4f). Collectively, these results suggest that the MERS-CoV antigen protein displayed on the surface of the viral vector appears to induce a stronger Th1-biased immune response than that on the bacterial vector.

### 3.4. Antigen-Specific T-Cell Immune Responses

**Figure 5:**
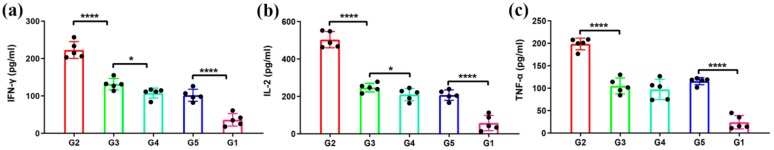
Immunization induced a significant cytokine response in mice. In order to evaluate antigen-specific T-cell responses, splenocytes were harvested from five mice in each group and were stimulated by MERS-CoV peptide S291 for 24 h. The level of secreted IFN-γ (**a**), IL-2 (**b**), and TNF-α (**c**) in the supernatants was detected by mouse ELISA kits. The data are shown as the mean ± SD and were analyzed by Student’s *t*-test (* *p* < 0.05, **** *p* < 0.0001, *n* = 5).

Next, in order to analyze the MERS-CoV specific T-cell immune responses, we detected the levels of secreted IFN-γ, IL-2, and TNF-α from the splenocytes after re-stimulation with MERS S291 peptide. The levels of IFN-γ and IL-2 in G3 were significantly higher than those in G4 and G5, suggesting that 1 µg of antigen protein on the bacterial vector is sufficient to induce potent IFN-γ/IL-2-expressing T-cell responses, whereas the levels of IFN-γ, IL-2, and TNF-α in G2 were remarkably higher than those in G3–G5 at 7 weeks post-immunization (w.p.i.) (Figure 5). These results indicate that MERS-CoV antigen protein displayed on the viral vector induces a superior cellular immune response than that on the bacterial vector.

## 4. Discussion

MERS-CoV continues to infect humans, causing morbidity and mortality since several outbreaks in 2012 that have caused a potential global MERS-CoV pandemic. However, no prophylactics and therapeutics available to protect against MERS have currently been licensed, although researchers have developed and tested several MERS-CoV vaccine candidates in many animal models. Each of these candidate vaccines has several advantages and challenges, such as preexisting immunity, immunogenicity, and cost-effectiveness. Here, we have developed two promising MERS-CoV vaccine candidates using two different platforms. One is the recombinant RV expressing MERS-CoV S1 protein fragment, and the other uses GEM particles as a vector to display the MERS-CoV RBD protein fragment. In this study, we compared the humoral and cellular responses in a BALB/c mouse model. The results indicate that the RV/MERS vaccine induces rapidly neutralizing antibody and stronger T-cell immune responses than the MERS-CoV BLP vaccine. However, the MERS-CoV BLP vaccine can induce a higher level of neutralizing antibodies than that of the RV/MERS vaccine.

Two unique aspects can be highlighted in our study. The first is about the RV vector, besides the MERS-CoV, which has been proven to be successfully utilized for many other emergent infectious diseases, such as the Ebola virus (EBOV) [27], Lassa virus (LASV) [28], Marburg virus (MARV) [29], human immunodeficiency virus type 1 (HIV-1) [30], and lymphocytic choriomeningitis virus (LCMV) [31]. Additionally, the Ebola virus vaccine is close to clinical trials [29]. Notably, chemically inactivated rabies virus vaccines have been widely used for vaccination of humans, dogs, cats, and ferrets, which shows that they are safe for humans and animals [32]. Therefore, the inactivated rabies virus-based vaccine is also safe and can induce neutralizing antibodies against the target pathogen. Here, the results show that the inactivated rabies virus vectors have very safe and immunogenic profiles in animals and highlight their promise for vaccine development.

The second unique aspect of this study is the GEM-PA system, a novel surface display system, which is a flexible, effective, cost-effective, and easy-to-handle alternative for heterologous proteins to be displayed on the GEM particles [33]. The GEM particles are from the *Lactococcus lactis* boiled in acids and mainly contain the bacterial-shaped peptidoglycan spheres, lacking other cell components [34]. Additionally, *Lactococcus lactis* has been safely applied in foods in recent years [35]. As such, the GEM-based vaccine is recognized as being safe. Furthermore, the GEM particles have been proven to enhance immune response as a carrier displaying the antigen with high density or adjuvant in vaccine development [36,37,38,39]. GEM particles can also improve the humoral and cellular immune response by activating the maturation of the dendritic cells, enhancing the antigen-presenting capacity, and stimulating the DCs to produce cytokines [40]. Besides that, peptidoglycan, the main component of the GEM particles, is a toll-like receptor (TLR) 2 ligand [38,40,41]. Moreover, GEM particles can efficiently bind the antigen protein at room temperature to obtain the purified proteins with only a one-step centrifugation process [33]. It is convenient and economical to scale up in vaccine development. GEM particles can also bind to different antigen proteins in the same particles to develop multivalent vaccines [42]. The system has successfully displayed a variety of heterologous proteins from pathogens, showing strong antigen-specific immune responses with parenteral vaccination or mucosal vaccination, including viruses, bacteria, and parasites [38,43,44,45,46]. In our study, the results suggest that the RBD protein of MERS-CoV displayed on the GEM particles could induce strong humoral and cellular immune response even when the dose is as low as 1 µg.

The two vaccine platforms in this study have their own advantages and disadvantages. The RV-based MERS-CoV vaccine platform can rapidly induce antibody immune response and higher cellular immune response than the GEM-based vaccine platform. However, there is a risk of incomplete inactivation in the process of inactivating the RV/MERS. Additionally, RV/MERS vaccine needs to be purified through many purification steps. These factors must be considered in vaccine production. Antigen protein density and distribution on a particle are two key parameters in eliciting an efficient immune response in vaccine design; a high density and ordered antigenic array displayed on a particle makes binding events between the host B-cell surface immunoglobulins and the particle occur more easily, which is an important step in inducing sequential immune response [47]. The GEM-based vaccine platform could display antigen protein in a high density, while the RV-based vaccine platform not only displayed the antigen protein but also expressed the RV GP protein. This may be why the GEM-based vaccine of MERS-CoV induces a much stronger humoral immune response in mice than that of the RV-based vaccine. Micro-particles could promote a humoral immune response, and nano-particles are much easier to induce a cellular immune response [48,49]. The size of the RV/MERS virion is about 167 nm, and the diameter of the MERS BLP particles is about 2161 nm. This may be a critical factor in inducing a different cellular immune response by two different vectors of vaccine platforms. Specific molecules of microbial origin, as intrinsic innate immune triggers, are endowed with adjuvant activity, such as lipopolysaccharide (LPS), bacterial lipopeptides, deoxyribonucleic acid (DNA), ribonucleic acid (RNA), and other conserved molecules [50]. The receptors for these molecules are the mammalian Toll-like receptors (TLRs). Members of the TLRs have been confirmed on the cell surface membrane (TLR1/2/4/5/6/10) and on the membrane of endosomes (TLR3/7/8/9) [51]. Peptidoglycan, the main component of the GEM particles, is a TLR 2 ligand which is an activation of TLR-2 receptors in human peripheralblood mononuclear cells inducing a Th1-type immune response [52]. Single stranded RNA, the component of inactivated rabies virus, is a TLR 7 ligand [51,53]. TLR7/8 agonists have shown Th1 polarizing effects on the immune response. Different receptors play an important role in delivering different signals to the host cells. The activated antigen presenting cells (APCs) precisely define the nature of the perceived danger and send this information to the secondary lymphoid organs, inducing relevant adaptive immune response. This may be another ignored factor of inducing a different immune response by the immune system thinking that the danger is from a virus infection or a bacterial infection. The immune system is triggered by a combination of events and stimuli in vaccine immunology. The compound adjuvants containing the emulsion and PolyI:C (TLR 3 ligand) may be another assignable cause. The speed of the antibodies’ production and cellular immune response needs to be further improved in future study of the GEM-based vaccine platform. To solve the problems, we can try to screen optimum adjuvants for the vaccine or co-displaying protein-adjuvants on GEM particles, such as flagellin (TLR 5 ligand). Furthermore, the GEM-based vaccine platform may be more suitable to develop mucosal vaccines considering that *Lactococcus lactis* has been safely used as a probiotic in our foods. Understanding the modes of action of the GEM-based vaccine platform will allow us to design a much safer, cost-effective, and protective vaccine with the desired immune response.

The goal of this study was to test vaccine vectors through a head-to-head comparison of immunogenicity of two MERS-CoV vaccine candidates as they might be used in real vaccines for humans or animals. This study provides an important reference for selecting a suitable vaccine platform in developing the MERS-CoV vaccine or other special pathogens in further study.
